# Supplementary material for: Are carbon emissions trading and green financial instruments synergistic? -Comprehensive quantitative research based on content analysis
Source: PLoS One. 2024 Mar 7;19(3):e0298601. doi: 10.1371/journal.pone.0298601 (PMC10919601; doi:10.1371/journal.pone.0298601)
Supplement: S1 Data — (ZIP) [file pone.0298601.s001.zip › Data/地级市Z_F工作报告.zip!/┴╔─■/╡ñ╢1⁄2╩╨/└┤╫╘╣1⁄2╓┌║┼í╛┬φ┐╦╩2╛▌═°í┐_2015.docx]

各位代表：

我代表市政府向大会报告工作，请予审议，并请市政协委员和各位列席人员提出意见和建议。

一、2014年主要工作回顾

一年来，在市委的正确领导下，在市人大及其常委会和市政协的有力监督、支持下，市政府带领全市人民，坚持稳中求进的总基调，积极应对经济持续下行压力，努力克服各种困难，着力稳增长、调结构、惠民生、促发展，保持了经济增长缓中有进、稳中向好，社会和谐稳定。预计地区生产总值1180亿元，比上年增长5.9%；固定资产投资903亿元，下降10%；公共财政预算收入实际完成126亿元，下降7.6%；预计城镇常住居民人均可支配收入22932元(新口径)，增长9.6%；农村常住居民人均可支配收入11619元(新口径)，增长11%；居民消费价格指数控制在1.6%。

(一)全力以赴稳增长，保持了经济持续健康发展

强化了全市经济运行的监测调度，积极应对严峻复杂的经济形势，经济结构调整进一步加快，实现了经济稳定增长和质量提升。

狠抓大项目落地和开工建设，一批重大基础设施项目的建设对投资产生了积极的拉动作用。辽东世贸城等143个亿元以上项目实现了开工建设，沈丹客运专线、丹大快速铁路征地动迁全部完成，线下工程施工基本结束；新鸭绿江公路大桥按期竣工，达到了通车启用条件，配套口岸联检区主体工程完工；丹东港完成港口投资约100亿元，20万吨级矿石泊位投入使用；三湾水利枢纽工程建成，应急供水管线基本铺设完毕。

出台了系列扶持政策，进一步加强了对重点骨干企业的包扶和中小微企业的支持，促进了全市工业经济平稳增长。预计规模以上工业增加值增长5.5%；高新技术产品产值增长16%。新增规模以上企业40户、销售收入亿元以上企业30户。列入省重点考核的4个产业集群年销售收入均突破百亿元。14个重点工业园区中东港经济开发区、前阳经济开发区销售收入均突破百亿元。钰岩矿业高钙粉加工、汇洋公司硼铁分离等33个亿元工业项目开工建设。

农业基础设施不断完善，促进了农产品的丰收和有效供给,粮食产量首次突破百万吨，达到106.7万吨。预计蔬菜总产量114万吨，增长3.6%；肉蛋奶总产量54.5万吨，增长2.3%；水产品总量61.4万吨，增长1.5%。县域经济得到较快发展，主导产业不断做大做强，东港、凤城分别成为全国草莓、板栗第一县，宽甸成为全国人参生产基地县。农业产业化发展规模不断壮大，引进投资亿元以上农产品加工项目12个，规模以上农产品加工企业达到220户，农产品创汇值8.3亿美元，居全省第2位。

全力提升服务业发展水平，规划并已着手建设了20个规模较大、特色鲜明、功能完善的服务业集聚区。东北亚商贸城、兴隆大家庭、华美新天地等大型商贸项目主体完工。消费市场活跃，万达广场等特色街“夜经济”繁荣。新兴业态和新商业模式悄然崛起，首个电子商务产业园成立。预计社会消费品零售总额478亿元，增长12%。旅游业不断向好，全力打造了五大旅游精品工程,完成了辽宁(宽甸)生态旅游实验区省级生态县创建工作，成功举办了鸭绿江口国际观鸟节、河口桃花节等活动，江海游、赴朝游、休闲游、红色游持续火爆。注重旅游宣传和城市知名度的提升，我市被评为“中国十大最美旅游城市”。

进一步强化了财税金融的保障作用。向上争取各类专项资金比上年增加10亿元，公共财政预算支出完成206亿元，支出重点向改善民生、促进发展倾斜。新引进的盛京银行、金元证券等5家金融机构开业。欣泰电气在深交所创业板上市；辽宁参仙源参业成为我市第一家在新三板挂牌企业及全国首个人参加工上市企业。银行机构本外币贷款余额924亿元，增长17.2%，新增贷款连续5年超过100亿元，完成各类融资285亿元。

(二)多措并举扩大对外开放，发展活力日益增强

重点的新兴开发区域实现项目入驻和功能完善同步驱动。边境经济合作区扩区获批。丹东新区实施亿元以上项目41个，加密延时了公交线路，开通了循环公交车，公共自行车系统投入使用；新区综合医院一期基本建成；国门湾家居生活广场投入运营，新增服务类商业网点70余个。大孤山经济区起重机制造等6个亿元以上工业项目开工建设；主干路网等基础设施快速推进，宫屯一次变项目基本竣工，大洋河应急供水项目全面开工，污水和垃圾处理工程已经启动。丹东港港口吞吐量达到1.38亿吨，增长15%；临港产业中，大豆深加工二期工程建成投产，三期工程开工；2座5万吨级船台、1座3万吨级船坞建成,4艘大型工程船舶交付使用、10余艘在建。翡翠湾德丰时代广场项目开工，佳兆业房地产二期工程完成规划方案，黄海南厂拆迁和场地平整基本结束；游艇码头项目启动，其中浪头港区清理出可利用岸线近300米，虎山码头开始建设，鸭绿江航道实施疏浚。高新区获得省政府批准，编制实施了防护纺织品产业集群总体规划；重点项目生物基尼龙56实现开工建设;成立了辽宁首家中科院产业技术创新与育成中心，入驻项目12个。

经过不懈的争取，丹东被列入全国重点开发开放试验区，目前试验区实施方案已报省政府待批，试验区总体规划编制全面展开。围绕我市重点产业，强化了主题招商、一对一招商，促进了欧尚广场商业综合体等一批重大项目落地。外贸出口稳中有升，丹东被海关总署批准成为对朝出境加工试点地区，有力推动了我市加工贸易转型升级；东港水产品养殖基地提升为国家级出口基地。第三届中朝经贸文化旅游博览会成功举办。丹东机场新建航站楼投入运营，开辟了新的航线。

(三)千方百计做好民生工作，各项实事得到较好落实

在市政府承诺办好的民生实事中，更加注重老城区的改造提升和群众切身利益的保障提高，不断弥补公共服务“短板”和基础设施“欠账”。胜利街、江城大街、东泰路等重要路段改造完成，国桢路、兴一路等城市铁路道口全部拓宽，四号干线锦江山隧道工程正在加紧施工。新增城区停车位2000多个。

完成了花园河等城市内河清淤工作。改造农村无害化卫生厕所1.7万座，新建和改造城市标准化水冲公厕、旱厕59座。完成了部分城市地下管网、住宅小区自来水二次加压泵站的改造任务。

大力推进金山热电城市集中供热联网，新增联网面积420万平方米，停用燃煤锅炉65台，供暖质量明显改善。

保障性安居住房基本建成6093套，新开工5660套。全市最大的工矿棚户区——五龙金矿棚户区改造工程竣工开园。

新增城镇就业6万人，登记失业率控制在4.5%以内。社会保险扩面征缴的年度任务超额完成，养老保险、城乡低保、五保对象、新农合标准均有提高，妥善解决了2000多名失地农民和1.5万名厂办大集体困难职工基本养老和医疗保险问题。完成了农村3.8万贫困人口的脱贫任务。

“四城联创”取得重大突破，我市荣获全省文明城市标兵称号，并取得全国文明城市提名。市容环境卫生综合整治成效明显，私搭乱建、马路工厂、交通违法、环境污染等得到治理。乡镇环境卫生整治走在全省前列，宜居乡村建设得到省委、省政府充分肯定，河口村等一批村镇获得国家级、省级荣誉称号，省委、省政府给予专项奖励资金2.2亿元。

(四)大力推进社会事业，公共服务水平逐步提升

92所学校通过了省义务教育标准化验收，12所新建公办幼儿园竣工，中小学全面实施阳光分班，职业教育创新转型深入开展。

成功承办了省第12届运动会，筹办工作和赛事组织扎实有序，安保工作严密到位，实现了“零事故、零差错、零失误”，办成了一届文明节俭、精彩热烈、开创新风的体育盛会。

公共文化服务扎实推进，创作了一批有影响的文化精品，话剧《你是一个好人》等3部作品荣获了省“五个一工程奖”，百姓春晚得到群众好评，天桥沟红色文化产业基地项目获得上级资金支持，文化惠民与群众文化需求实现了有效对接。

国家11类基本公共卫生服务项目得到落实，东港、凤城公立医院实施了取消药品加成试点改革，基本药物制度深入实施。全面落实了“单独二胎”政策。市残疾人康复中心正式投入使用。

精神文明建设深入实施，志愿者服务工作取得新成效，全市有21万市民注册成为社会服务志愿者。

积极推进碧水、蓝天、青山工程，加强节能减排，全市生态环境进一步改善，鸭绿江和北黄海近岸海域水环境质量全部达标，环境空气质量明显优于省内其他城市。

对各类犯罪保持高压严打态势，防控体系进一步完善。

开展了打击走私贩毒、非法捕捞等专项整治行动，通过增加高清摄像头、安装船只定位系统等方法提高了科技管边控边能力，保持了边境地区和谐稳定。民兵预备役和双拥工作取得新成果。妥善解决了一批事关群众利益的信访问题。应急管理体系不断完善，安全生产形势持续稳定好转，平安丹东建设取得明显成效。

(五)不断深化改革，政府自身建设进一步加强

大力实施简政放权，不断深化行政审批制度改革，深入开展行政职权事项清理，进一步理顺了近年来向城区、经济区下放的58项管理权。稳步推进政府机构改革，完成工商、质监、食品药品监督管理体制改革及卫计委组建任务，初步实现对食品、药品安全在生产、流通、消费环节的统一监管。工商登记制度改革激发了市场活力，新登记公司制企业和认缴注册资本分别增长104%和268%。

结合党的群众路线教育实践活动，认真整治政府系统在“四风”方面存在的突出问题，有效遏制了“三公”经费和办公用房等方面的浪费，特别是在清房工作中，采取市委、市政府集中办公等措施，清理办公用房面积约17万平方米。

自觉接受市人大及其常委会的监督和市政协民主监督，认真听取各民主党派、工商联、无党派人士和人民团体意见。办理市人大代表建议、政协提案677件，见面答复率和办结率均达到100%。

外事、侨务、台务、民族宗教、防震减灾、老龄等工作也取得了新成果。这些成绩的取得是市委正确领导和市人大、市政协大力支持的结果，是全市上下攻坚克难、共同努力的结果。在此，我代表市政府向全市各族人民，向各民主党派、工商联、各人民团体、各界人士以及驻丹单位和驻军部队表示衷心的感谢并致以崇高的敬意！

各位代表，在肯定成绩的同时，我们也清醒地认识到，全市经济社会发展还存在一些不容忽视的问题，主要表现在：一是经济下行压力依然很大，主要经济指标增速放缓，尤其是工业经济下行趋势明显，一些行业企业生产经营比较困难；二是投资增速减弱，亿元以上项目投资额和在建项目均有所下降，重大项目尤其是工业大项目的引进仍然没有大的突破；三是沿江沿海经济带发展不均衡，新开发区域推进不快；四是公共财政收入呈现负增长，虽然有主动挤掉水分、做实税收的因素，但收入有所减少，进一步加大了刚性支出和政府债务偿还的压力；五是政府系统“四风”问题虽已得到有效遏制，但机关慵懒散、慢作为等现象仍然存在。这些问题已引起我们的高度重视，在今后的工作中，将采取切实有力的措施加以解决。二、2015年经济社会发展目标任务和重点工作

各位代表，2015年是全面深化改革、扩大开放的重要一年，也是“十二五”规划收官、做好“十三五”规划编制的重要之年。随着经济下行压力的不断增大，其滞后效应将会对今年经济发展带来很大影响，我们面临形势的复杂性将前所未有、严峻性前所未有、困难和挑战也将前所未有。但是，我们充分认识到有压力必然有动力，有困难必然有机遇。党中央、国务院和省委、省政府提出了稳中求进的要求，制定了新常态下实现健康发展的各项措施，必将引导经济不断向好。因此，我们必须增强必胜的信心，凝聚迎难而上的士气，拿出敢于担当的勇气，释放敢闯敢拼的锐气，在严峻形势下破除坚冰，寻求突破。为此，市政府确定今年全市经济社会发展的总体思路是：认真贯彻落实中央、全省经济工作会议精神，按照市委十一届九次全会的要求，坚持稳中求进工作总基调，抢抓东北振兴新十年、沿边地区开发开放、改革激发内生动力三大机遇，主动适应经济发展新常态，实施“以港兴市”战略，以提高经济发展质量和效益为中心，把改革贯穿于经济社会发展各个领域、各个环节，坚定不移地扩大开放，优化调整产业结构，统筹新老城区一体化发展，拓展沿江沿海开发空间，保持经济平稳健康发展、社会和谐稳定，努力在新一轮振兴发展中实现新突破。

今年全市经济社会发展的主要预期目标是：地区生产总值比上年增长6%；固定资产投资增长5%；公共财政预算收入增长2%；社会消费品零售总额增长11%；城镇常住居民人均可支配收入增长7%；农村常住居民人均可支配收入增长7%；居民消费价格指数控制在3%以内。为实现上述目标和任务，我们将全力抓好以下重点工作。

(一)以优化城市空间布局助推沿江沿海区域发展

整合新区、翡翠湾、临港区域、高新区、大孤山等新开发区域，推动沿江沿海一体化开发建设、联动发展，同时辐射和带动城市北部地区，逐步形成有利于城市发展的空间布局。

新区作为丹东未来发展的引擎，以扩区为契机，加快转型升级、产城融合，重点实施项目规划与土地一级开发，拓展区域建设新平台，黄海新能源汽车、纳诺能源装备、粮食加工产业化等项目实现竣工投产；进一步加快新区功能配套，多措并举繁荣新区，实现国际金融会展商业综合体、新区大型购物超市等项目开工建设，中科院幼儿园开班办学，新区医院启动运行，积极推进辽东学院搬迁工作。

大孤山经济区全力实施路网水电等配套要素工程，提升招商引资基础条件，实现金利冶金、明清仿古一条街等项目开工建设。

丹东港临港产业在加快造船与海工装备制造业、现代农业深加工的基础上，加快推动有机农产品产业、冷链物流园区、进口木材物流园区等项目的实施。

翡翠湾重点推进德丰时代广场、游艇码头、柏林大道等项目建设，加快土地收储，积极招商引资。

高新区力争实现生物基尼龙56项目一期工程投产，纺纱、医疗防护等项目开工建设，逐步向纤维、纺织、染整、服装等产业链延伸。

(二)以重大基础设施建设带动项目落地和实施

推进重大基础设施项目建设。竣工两条高铁。千方百计创造条件，确保沈丹客专8月末通车，丹大快铁9月末通车，使丹东尽快融入沈阳、大连一小时经济圈。

新建三条高速公路。启动宽甸至本溪碱厂高速公路项目，先行实施动迁工作，开辟沈阳及周边地区到宽甸旅游新的便捷通道。开工建设大东港疏港高速公路，提高港口集疏运能力，为做大港口和临港产业创造有利条件。新增丹大高速公路前阳立交出口，先行建设10公里连接线，推动前阳经济区加快发展。

改造两个城市出口。对花园路和金山大街进行拓宽改造，缓解出城交通拥挤状况，增加城市北部地区发展纵深。

增加新区出口。打通新鸭绿江公路大桥、黄金坪中方配套区与201国道的两条连接线，不断提高新区对外通达度。

开展新老城区公共交通体系可研论证。加快推进新老城区有轨电车、快速公交及水上巴士等综合交通体系项目的可研论证，为尽快形成新老城区交通一体化奠定基础。

加快港口建设。丹东港大东港区继续推进大型专业化泊位项目建设，启动30万吨级航道工程；海洋红港区推动5万吨级通用泊位及相关工程建设。

实施抗美援朝纪念馆改扩建工程。力争上半年获国家发改委立项审批，并开工建设，同步完善史料和功能，扩大其作为国家级爱国主义教育基地的影响力。

抓好“气化丹东”工程。实施大连至丹东的天然气“西气东输”项目，让更多群众用上安全高效清洁的天然气。

推进金山热电二期工程。做好金山热电二期工程前期工作，让集中供热惠及更多市民。

启动垃圾处理场和污水处理厂建设。采取政府和社会资本合作等多种投融资模式，建设无害化垃圾处理场，逐步解决城市垃圾污染问题。同时启动新区污水处理厂建设。

破解项目落地开工难题。实施目标责任制管理，明确责任人、时间进度和保障措施，市县(区)两级政府领导每人负责一个重点园区和两个以上重大项目，重点解决项目签约落地难、开工难问题，做好动迁工作，减少审批环节，提高审批效率。全力推进大孤山约翰森汽车配件、凤城王老吉系列饮料等100个亿元以上重点项目，其中新开工70个，续建20个，开展前期工作10个。

强化土地储备和整理。加大资金投入，通过道路改造、河道治理等途径，储备和整理更多的土地资源，为城市发展、项目落地提供要素保障。

做好“十三五”规划编制和东北振兴新十年项目储备。结合我市目前发展实际，科学编制“十三五”规划，用足用好国家重点开发开放试验区和东北振兴新十年各项政策，抓好重大民生、基础设施、新兴产业和传统产业升级改造等项目谋划、筛选、包装和储备。

(三)以开发开放试验区实施引领对外开放

加快丹东重点开发开放试验区建设。力争试验区规划和建设实施方案上半年获得国家批准，并逐步规划建设丹东综合保税物流园区、跨境经济合作区、国际港口物流区、现代临港产业区、特色优势产业区、国际都市服务区和边海生态旅游区。同时积极向上争取早日享受沿边开发开放相关政策。

提升外贸出口和利用外资水平。外贸出口着力巩固朝鲜、日韩等传统市场,积极开拓欧盟等新兴市场，争取汽车零部件和草莓出口基地升级为省级出口基地，利用电子商务、出口信用担保等平台，进一步促进出口。扩大丹东边贸企业对朝贸易人民币结算范围，启动互市贸易区和出境加工区建设。办好第四届中朝经贸文化旅游博览会。积极引进世界500强企业。加强对外经济技术合作，引导企业境外投资，推进有条件的企业海外上市。力争丹东机场开通临时口岸，增加至韩国的国际航线，加密国内航线。

抓好项目招商。紧盯国内外知名企业、上市公司、科技型创新企业，承接好“东北振兴央企辽宁行”项目，重点围绕产业配套和产业链延伸开展招商引资，引进带动力强、科技含量高、低能耗的大项目。强化主题招商，拓展以商引商，重点在旅游、温泉养生、电子商务、仓储物流等方面开展有针对性的项目对接。

(四)以科技创新促进工业产业升级

做强骨干企业。加大政策引导力度，以支柱产业、龙头企业为重点，助推骨干企业壮大规模，增强内生动力，带动行业和产业链共同发展，力争全年新增销售收入超亿元企业10户。

扶持小微企业。全面落实各项政策，发挥企业监测服务平台作用，注重生产要素的倾斜，积极帮助小微企业开发产品、开拓市场、开展融资，力争新增主营业务收入超2000万元企业30户。

促进企业转型升级。加强产学研合作，以产业技术创新与育成中心为载体，建设产业技术创新平台，推动专业孵化器建设，解决企业在人才、技术等方面难题，切实推动科技成果转化。抓好100户国家、省、市级创新示范企业培育，100项重点新产品试制投产，100项重点新技术、新工艺项目实施，切实提升企业创新能力。

提升产业集群规模。进一步加强研发检测、工业设计、融资服务、专业展会等公共服务平台和企业激励体系建设，为产业集群发展、核心园区建设提供保障，尤其抓好列入省政府考核的产业集群建设，推进不同规模产业集群的梯次发展，积极培育满族医药、工艺美术等新兴产业集群。

做好服务和要素保障。完善工业经济运行监测、用电监测、生产要素保障体系，强化企业水、电、气、资金等方面的协调供给，积极帮助企业克服下行压力，逐步实现向好发展。

(五) 以新型城镇化建设拉动农村经济发展

加快推进新型城镇化建设。全面落实中央和全省城镇化工作会议精神，以人为核心，以改革创新为动力，不断优化城镇布局，探索以市场化运作模式加强县城、重点镇和特色镇建设的新路子，提高城镇建设水平和综合承载力，促进产城融合发展，加快农业转移人口市民化步伐，建设“环境整洁、设施完善、生态优良、传承历史、富庶文明”的宜居乡村。

不断壮大县域经济。以市场需求为导向，大力推进农业产业化经营，抓龙头带农，抓科技兴农，抓基地扶农，抓市场活农，促进传统农业的优化升级，提高农业的整体效益。依托县域独有的资源优势、产业优势，着眼于精深加工，延伸产业链条。加快工业园区建设，实现产业聚集，壮大县域产业集群规模。

大力发展特色农业。稳定粮食产量，抓好“菜篮子”工程，加速发展设施农业，优化蔬菜生产布局，进一步提高农产品供给能力和安全水平。加快培育农业产业化龙头企业，新增年销售收入亿元以上企业5户，新增投资亿元以上产业化项目10个。抓好农业出口示范基地建设，确保农产品出口额保持8%以上增长。加快特色农业基地和“一县一业”发展，做好品牌宣传，集中力量搞好东港国家级现代农业、宽甸生态旅游农业、凤城林牧农业、城郊都市农业等示范区。强化农田水利和林业生态建设。

(六)持续提升现代服务业发展水平

促进商贸物流等产业发展。重点抓好服务业集聚区建设，使其成为承接服务业项目的主要平台和促进服务业发展的强劲动力。加快电子商务产业园开发，吸引更多的电商企业入驻。抓好商品市场和物流市场建设，促进消费市场繁荣。承接沈丹客专、丹大快铁开通带来的大量人流、物流、信息流、资金流，促进商贸物流企业形成规模化经营。充分发挥我市的区位、环境、交通等优势，大力发展社会养老服务业，推进社区养老服务体系建设。保持房地产业健康发展。

推动旅游业整合做精。由市级统一规划，做好全域旅游景点的高质量、高标准开发和旅游产品的规划设计。研究制定两条高铁开通后引导、承接游客的对策。依托东北东部铁路、丹东机场和“东方明珠号”航线，将丹东打造成东北东部城市赴韩国、朝鲜等旅游首选中转站。以虎山长城、断桥、新鸭绿江公路大桥景点为支撑，开通鸭绿江景观全线游。深入开展辽宁(宽甸)生态旅游实验区建设。加快推进甲午海战博物馆、凤凰山景区综合改造、宽甸朝鲜族风情温泉度假村等项目建设，培育独特的旅游文化品牌。加强温泉资源规划管理，发挥资源优势，做强温泉旅游产业。通过有序开发，促进观光型旅游向休闲度假、养生养老等多元化旅游转型。创新旅游宣传促销模式，丰富观鸟节、桃花节、枫叶节、采摘节等特色节庆活动的内容。加强区域合作，推进智慧旅游工程建设。开发具有地方特色、品位较高的旅游纪念品。

加快金融服务创新。以增加中小企业融资为核心，不断拓宽小贷公司、担保公司和企业上市等多种融资渠道。力争企业在主板市场申报1户、新三板市场挂牌1户；新增或引进金融机构2家、小贷公司5家。全年确保完成各类融资额210亿元。

(七) 积极推动重要领域改革

深化国企改革。准确掌握全市国有企业详细情况，采取多种途径，解决企业遗留问题，重点抓好经营困难企业的改革，妥善做好其资产处置和职工安置工作。

实施农村改革。抓紧落实中央确定的农村集体土地所有权、承包权、经营权三权分置的改革任务，引导土地经营权有序流转。重点做好农村土地征收、集体经营性建设用地入市、宅基地制度改革的准备工作。

抓好财政改革。严格执行新《预算法》，形成一套较完善的现代预算制度。提高财政收入质量，增强财政保障能力。积极发挥财政杠杆作用，压缩一般支出，整合财力投向重点民生工程和项目建设。加强政府债务管理，严控新增债务，通过用政府债券置换原债务等措施，降低还债成本。

探索投融资体制改革。逐步改变公共基础设施项目单纯依靠政府投资的方式，鼓励和引导社会资本参与城市基础设施建设。

加快医疗卫生改革。深化公立医院改革，做好乡镇卫生院与计生服务中心整合工作。

推进现代公共服务体系改革。继续实施简政放权，做好198项行政职权的取消和下放工作。深化政府机构改革，修订完善市政府各部门“三定”规定，做好各县(市)区改革的指导。开展公车改革。落实县以下机关公务员职务与职级并行制度。推进市、县(市)区和乡镇三级行政服务体系建设，构建网上审批平台，不断提高审批效率。整合完善投诉举报平台，畅通民意诉求渠道，进一步解决群众办事难问题。

(八)全力做好民生工作

提高社会就业和保障能力。实现新增城镇就业5万人，城镇登记失业率控制在4.2%以内。继续完善社会保障体系，加快推进社保业务经办“五险合一”。保障好城乡低保对象等困难群体的基本生活。完成3.5万农村贫困人口脱贫任务。

改善市政道路条件。实施黄海路、大众路、锦山大街鹏欣水游城段等重点街路改造，继续推进四号干线建设，进一步完善城市路网功能。

加快保障房建设。推进城市棚户区改造，力争全市基本建成保障房8000套，新开工5000套。

继续实施老城区基础设施改造。完成20个1万平方米以上老旧小区改造任务。加快重点街路老旧燃气管网和住宅小区自来水二次加压泵站改造。开展生活垃圾分类试点，逐步建立和完善覆盖城乡的垃圾收运体系。启动大沙河以东至九连城地区市政雨污分流管网建设。做好青年湖等城市公园综合改造和城区绿化美化亮化工作。

抓好水利工程建设。进一步加强水源地保护。积极应对去冬今春所面临的严重旱情，启动抗旱应急预案，加快推进城市应急供水保障工程建设，实现三湾水利枢纽工程尽快向丹东市区供水。全力做好各县(市)区、经济区水库蓄水，确保城乡群众生活用水和灌溉用水需要。新解决6万农村人口饮水困难问题。在汛期前完成鸭绿江界河赵氏沟段和庙沟段护岸工程。继续实施大洋河、大沙河等河流综合治理改造。

深入开展卫生环境综合整治。加快美丽丹东建设，巩固“四城联创”成果，确保全国文明城市测评位居全省前列。深入推进乡镇环境卫生整治，逐步将整治成果向村屯延伸。强化农村基础设施建设，提高村镇公共服务水平，抓好秸秆禁烧和综合利用。继续抓好市容环境卫生综合整治，多手段、全方位整治私搭乱建、占道经营等违法违规行为，以及造脏、扰民、污染、妨碍交通等城市乱象。继续做好城乡卫生厕所改造工作。

加强生态文明建设。深入推进生态市创建工作，持续实施碧水、蓝天、青山工程，严把项目环保准入关，逐步淘汰高污染企业，强化环境污染治理，严厉打击环境违法犯罪，确保域内良好的生态环境。

(九)全面推进社会事业繁荣发展

抓好教育、文体、卫生等工作。推进基础教育强县(市)区建设，增加公办幼儿园数量，强化职业教育，实现各级各类教育的协调发展。坚持中小学阳光分班，有效遏制择校入学，坚决治理乱补课、乱办班等教育热点问题，加强校园周边治安、交通、饮食、卫生等综合治理。高度重视人才工作，完善和落实政策，加大高端人才的培养、引进和使用力度。大力发展文化、体育产业，努力创作文化精品，积极申报凤城、宽甸国家级“辽东文化生态保护示范区”，加快市文化宫维修改造，提升城市社区书屋建设水平；完成桃源多功能运动场、月亮岛体育广场、三马路青少年体育活动中心项目，推进社区(乡镇)全民健身场所建设。深入实施基层医疗机构基本药物制度，加快卫生信息化建设，提高公共卫生服务质量，落实好计划生育政策。加强市场监管，打击制假售假行为，构建保障食品药品安全的长效机制。

保持边境地区和谐稳定。提高国家安全意识，严打各类违法犯罪活动，进一步深化治安防控体系和规范抵边建设。深入实施“爱民固边模范市”创建活动，不断强化党政军警民“五位一体”的管边控海新机制。切实抓好安全生产各项责任和制度的落实，有效减少一般事故，遏制较大事故，杜绝重特大事故的发生。依法规范信访秩序，认真解决群众合理诉求。进一步推动军民深度融合发展，争创全省第八轮、全国第七轮双拥模范城。

(十)切实加强政府自身建设

坚持依法行政，建设法治政府。秉承依法治国理念，严格依法行使权力、履行职责，建设“法治丹东”、“诚信丹东”。自觉接受市委领导，认真执行市人大及其常委会的决议、决定，积极支持市政协履行职能。建立行政机关内部重大决策合法性审查机制，把公众参与、专家论证、风险评估、合法性审查和集体讨论作为重大决策的必经程序。强化行政执法监督，有效规范行政许可、行政处罚、行政征收、行政收费等行为。全面推进政务公开，努力使决策、执行和结果公开透明。加快社会信用体系建设，做好信用数据的征集和使用。加大普法、依法治理和法律援助工作力度。

深入改进作风，建设务实政府。继续落实好中央和省委、市委关于改进工作作风、密切联系群众的各项制度。深入实际、基层和群众，切实提高政府的主动服务意识。进一步精简会议、文件简报，改进会风、文风。严格控制“三公”经费支出，禁止新建楼堂馆所。

秉承清廉从政，建设廉洁政府。扎实推进惩治和预防腐败体系建设，努力从源头上防治腐败。加强监察和审计工作，完善公共资源交易系统，加大对违法违规问题查处力度，强化对权力运行的监督制约。抓好软环境建设，重点治理公职人员不作为、不担当行为，建立监管长效机制。

各位代表，我市全面深化改革和新一轮扩大开放的号角业已吹响，我们将在党的十八届三中、四中全会精神的指引下，义不容辞地肩负起全市人民交给的重担，坚定不移地落实全市人民赋予的责任，进一步坚定信心，振奋精神，开拓进取，攻坚克难，为丹东各项事业的发展、人民的幸福平安和谐做出最大的努力！
